# Emotional blunting in patients with depression. Part III: relationship with psychological trauma

**DOI:** 10.1186/s12991-022-00395-1

**Published:** 2022-06-21

**Authors:** Michael Cronquist Christensen, Hongye Ren, Andrea Fagiolini

**Affiliations:** 1 Medical Affairs, H. Lundbeck A/S, Ottiliavej 9, 2500 Valby, Denmark; 2 Division of Psychiatry, Department of Molecular and Developmental Medicine, University of Siena School of Medicine, Siena, Italy

**Keywords:** Depression, Emotional blunting, Functioning, Functioning Assessment Short Test, Oxford Depression Questionnaire, Psychological trauma

## Abstract

**Background:**

This international online survey investigated the experience and impact of emotional blunting in the acute and remission phases of depression from the perspective of patients and healthcare providers (HCPs). This paper presents data on the history and severity of psychological trauma and its potential impact on emotional blunting in major depressive disorder (MDD); differences between patient and HCP perceptions are explored.

**Methods:**

Patient respondents (*n* = 752) were adults with a diagnosis of depression who were currently taking antidepressant therapy and reported emotional blunting during the past 6 weeks. HCPs provided details on two eligible patients: one in the acute phase of depression and one in remission from depression (*n* = 766). Trauma was assessed using questions based on the Childhood Trauma Questionnaire; emotional blunting was assessed using the Oxford Depression Questionnaire (ODQ). Multivariate regression analyses were applied to examine the relationship between trauma and ODQ score.

**Results:**

A history of any childhood or recent traumatic event was reported by 97% of patients in the self-assessed cohort and for 83% of those in the HCP-assessed cohort (difference, *p* < 0.01). Patients were more likely than HCPs to feel that this trauma had contributed to their/the patient’s depression (58% vs 43%, respectively; *p* < 0.01) and that the depression was more severe because of trauma (70% vs 61%, respectively; *p* < 0.01). Emotional blunting was significantly worse in patients who reported severe trauma than in those who had not experienced severe trauma (mean total ODQ score, 90.1 vs 83.9, respectively; *p* < 0.01). In multivariate regression analyses, experiencing both severe childhood and recent trauma had a statistically significant impact on ODQ total score (*p* = 0.001).

**Conclusions:**

A high proportion of patients with depression and emotional blunting self-reported exposure to childhood and/or recent traumatic events, and emotional blunting was more severe in patients who reported having experienced severe trauma. However, history of psychological trauma in patients with MDD appeared to be under-recognized by HCPs. Improved recognition of patients who have experienced psychological trauma and are experiencing emotional blunting may permit more targeted therapeutic interventions, potentially resulting in improved treatment outcomes.

## Introduction

It is well documented that exposure to psychological trauma can have negative effects on mental health. A history of trauma (e.g., during childhood) has been shown to be associated with an increased risk of developing mental health disorders, such as depression [1–4], and patients with depression are significantly more likely to report exposure to traumatic life events than individuals without depression [5–7]. In the International Study to Predict Optimized Treatment for Depression (iSPOT-D), patients with depression were found to be significantly more likely than healthy control participants to report early-life stress—particularly interpersonal violation (emotional, sexual, and physical abuse) [7]. In all, 62.5% of patients with major depressive disorder (MDD) reported more than two traumatic events, compared with 28.4% of control individuals [7].

The COVID-19 pandemic has had a major psychological impact worldwide. Studies have shown increased prevalence of post-traumatic stress disorder, anxiety, and depression in the general population of many countries since the start of the pandemic, both in individuals who have contracted the virus and those who have not [8–17]. The psychological impact of the pandemic appears to be even greater in certain groups, such as patients with pre-existing mental health conditions [18, 19].

A history of psychological trauma has been shown to be associated with more severe disease in patients with depression [20–22], including poorer responses to antidepressant treatment [7, 22, 23] and a greater risk of suicide [24, 25]. In iSPOT-D, patients with MDD who had been exposed to abuse at 4–7 years of age were significantly less likely to achieve response or remission following treatment with a selective serotonin reuptake inhibitor (escitalopram or sertraline) or serotonin-noradrenaline reuptake inhibitor (venlafaxine extended release) than those who had not experienced abuse at this age [odds ratio 1.57 for response (*p* = 0.034) and 1.61 for remission (*p* = 0.032)] [7].

Emotional blunting is a common symptom in patients with MDD and is increasingly being recognized as an important factor preventing full functional recovery [26–29]. However, data on the association of psychological trauma with emotional blunting in patients with depression are lacking. Anhedonia—the inability to experience pleasure—is one aspect of emotional blunting, and is one of the core diagnostic criteria for a major depressive episode [30]. Patients with MDD and a history of psychological trauma have been shown to experience greater levels of anhedonia than those without such a history [31]. Emotional blunting or numbing is also common in prolonged grief disorder [32] and is a core defining feature of post-traumatic stress disorder, presenting as diminished interest in activities, detachment from others, and the inability to experience positive emotions [33, 34]. In patients with post-traumatic stress disorder, the presence of emotional numbing has been shown to be associated with increased symptom severity, functional impairment, and suicide ideation [34–36].

This online survey was undertaken to explore the experience of emotional blunting in patients with MDD in the acute and remission phases of depression from the perspective of both patients and healthcare providers (HCPs). Findings concerning the clinical characteristics of emotional blunting in patients with depression and the impact of emotional blunting on functioning and overall quality of life from the patient perspective have been reported in the first two papers in this series [37, 38]. The current paper presents data on the prevalence and severity of psychological trauma and its potential impact on the severity of emotional blunting in patients with depression who were experiencing emotional blunting; differences between patient and HCP perceptions are also explored.

## Methods

### Study design and participants

This was a quantitative, cross-sectional, observational study conducted by BPR Pharma (London, UK) in Brazil, Canada, and Spain between April 15 and May 18, 2021. The study design is described in detail in the first paper in this series [37]. The study was approved by an institutional review board (Veritas IRB, Montreal, QC, Canada), was conducted in accordance with the European Pharmaceutical Market Research Association (EphMRA) code of conduct, and adhered to General Data Protection Regulation (GDPR) and all local market laws regarding data protection. Participants were recruited through an existing online panel of consumers and healthcare providers. Participants had previously consented to participate in research; however, informed consent was also obtained specifically for this study.

Patient respondents were aged 18–70 years, had been diagnosed with depression by a physician, were currently taking a prescribed antidepressant, and reported experiencing emotional blunting in the last 6 weeks. Emotional blunting was confirmed by a validated screening question [39]: *‘Emotional effects of depression and treatment vary, but may include, for example, feeling emotionally “numbed” or “blunted” in some way; lacking positive emotions or negative emotions; feeling detached from the world around you; or “just not caring” about things that you used to care about. Have you experienced such emotional effects during the last 6 weeks?’ *Only patients in the acute or remission phases of depression were eligible for study participation. The acute phase was defined as: *‘A time when your symptoms are at their worst or most severe and for which you use antidepressant treatment.’* The remission phase was defined as: ‘*A time when your symptoms have improved significantly and you are already feeling better, but you may or may not still experience some minor symptoms. You are still taking antidepressant medication*.’ Quotas were imposed for patient age (≥ 50 years, 50%) and sex (female, 60%).

HCP respondents were psychiatrists (*n* = 226) or primary care physicians (*n *= 157) spending ≥ 75% of their time in direct patient care and prescribing antidepressants to ≥ 75% of their patients with depression. For psychiatrists, quotas were imposed on hospital and office settings (each 50%). Patients assessed by HCPs were required to be aged 18–75 years, diagnosed with depression, and receiving antidepressant medication. HCPs completed the survey for the last two eligible patients with whom they had a consultation: one in the acute phase of depression (defined as *‘A patient experiencing acute symptoms of depression that require antidepressant treatment’*), and one in the remission phase of depression (defined as ‘*The patient feels better and experiences a significant reduction in symptoms compared to other phases. Some residual symptoms may persist, but are significantly fewer in number and severity compared to other phases. The patient is still on antidepressant medication*’).

Patients and HCPs were unmatched (i.e., patients who participated in this survey were not the same patients as those described by the HCPs).

### Outcome measures

For both surveys, trauma questions were based on the validated Childhood Trauma Questionnaire [40]. Questions concerned: (i) childhood trauma (i.e., trauma occurring before the age of 17 years); and (ii) recent trauma (i.e., occurring within the last 3 years). Questions captured whether patients had experienced any of a range of different traumatic events during these two time periods, including: the death of a very close friend or family member; any upheaval between parents or with a spouse (e.g., their parents’ or their own divorce or separation); a traumatic sexual experience (e.g., rape or sexual assault); being a victim of violence (other than sexual); being extremely ill or injured; major change at work (recent trauma only); and any other major upheaval that may have significantly shaped their life or personality. Patients reported their personal experiences of childhood and recent psychological trauma; HCPs reported whether their respective patients had experienced these same traumas in childhood or recently. Patients also rated the severity of the traumatic event experienced on a 7-point numerical rating scale, where a score of 6 or 7 was considered indicative of severe trauma. HCPs were not asked to rate trauma severity.

Patients assessed the severity of their emotional blunting using the Oxford Depression Questionnaire (ODQ). This validated instrument for assessing emotional blunting in patients with depression comprises 26 questions covering five domains: general reduction in emotions, reduction in positive emotions, emotional detachment from others, not caring, and antidepressant as cause [39, 41]. The ODQ total score ranges from 26 to 130, with higher scores indicating more severe emotional blunting. Patients also completed the Functioning Assessment Short Test (FAST), a brief self-report instrument that is used to assess problems with daily functioning [42]. For the purposes of this survey, the period of recall for the FAST was ‘*during this acute or remission phase of depression*’. The FAST total score ranges from 0 to 72, with higher scores indicating greater functional impairment.

### Statistical analysis

The analysis population comprised all respondents who met the inclusion criteria and completed the online survey. Respondents who failed to complete the survey or who completed the survey much faster than average were excluded from the final sample. Any respondent who responded ‘don’t know’ to more than one item for any FAST domain was excluded from the analysis of that domain. For patients who responded ‘don’t know’ to just one item in any FAST domain, the mean score for the other items answered in that domain was used to impute the missing value (as per the scale guidance). Respondents with a missing score for any domain were removed from the calculation of FAST total score (a missing score would result from them having responded ‘don’t know’ to more than one item in that domain).

Data were analyzed separately for patients and HCPs. Data are presented descriptively using means and standard deviations for continuous variables, and frequencies and percentages for categorical variables. Comparisons were performed for continuous measures using *t *tests and for proportions using *Z* tests. For the patient-reported cohort, ODQ scores and FAST total score were analyzed according to the type of severe trauma reported (severe childhood, severe recent, or any severe).

Multivariate regression analysis was applied to assess whether a history of psychological trauma had an independent and significant impact on emotional blunting (i.e., ODQ total score) after controlling for other patient characteristics (demographics and symptoms of depression). Regression was assessed on: (a) demographic variables (age group, sex, education, country); (b) symptoms of depression (anxiety, physical symptoms, cognitive symptoms, mood symptoms); and (c) trauma type (severe childhood trauma only, severe recent trauma only, both severe childhood and recent trauma, neither). Variable sets (a), (b), and (c) were entered hierarchically as blocks. Block (a) was force-entered, with variables in block (b) and (c) entered only if they had a statistically significant effect (determined using backward and forward selection). Statistical significance determined by either method was considered valid.

Data were analyzed by The Stats People (Sevenoaks, UK) using MERLIN tabulation software and Microsoft Excel; significance was set at *p* < 0.05.

## Results

### Experience of trauma

Data were available for 752 self-assessed and 766 HCP-assessed patients (Table 1). Sociodemographic characteristics were generally similar in the two patient cohorts. However, the proportion of patients with a history of past or present drug or alcohol abuse was more than twice as high in the self-assessed cohort than in the HCP-assessed cohort, both overall and for each phase of depression (acute and remission). The proportion of patients with a history of any trauma was significantly higher in the self-assessed cohort than in the HCP-assessed cohort (97% vs 83%, respectively; *p* < 0.01).

**Table 1:** Patient demographics and prevalence of trauma (patient-reported and HCP-assessed cohorts)

	Patient-reported cohort (*N* = 752)	HCP-assessed cohort (*N* = 766)
Sex, *n* (%)		
Female	466 (62)^a^	484 (63)^a^
Age, years		
Mean (SD)	45 (12.5)	44 (13.1)
Depression phase, *n* (%)		
Acute	300 (40)	383 (50)^a^
Remission	452 (60)	383 (50)^a^
Ever addicted to drugs or alcohol, *n* (%)	156 (21)*	68 (9)
Acute	71 (24)	38 (10)
Remission	85 (19)	30 (8)
Any trauma, *n* (%)	732 (97)**	638 (83)
Childhood	650 (86)	327 (43)
Recent	691 (92)	579 (76)
Any severe trauma, *n* (%)	658 (88)	–
Childhood	515 (68)	–
Recent	562 (75)	–
Both childhood and recent	419 (56)	–
Trauma as reason for depression, *n* (%)	425 (58)^b^**	274 (43)^c^
Depression more severe due to trauma, *n* (%)	509 (70)^b^**	389 (61)^c^

HCPs were not asked to rate trauma severity

*HCP* healthcare provider, *SD* standard deviation

^a^By design

^b^*n* = 732

^c^*n* = 638

^*^p < 0.05  vs HCP-assessed cohort

^**^p < 0.01 vs HCP-assessed cohort

**Fig. 1:**
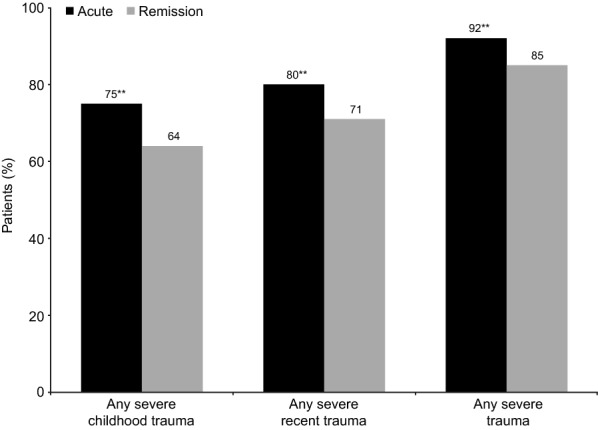
Proportion of patients reporting severe trauma by phase of depression (patient-reported cohort). Severe trauma = score of 6 or 7 on a 7-point severity scale. **p < 0.01 vs remission phase

**Fig. 2:**
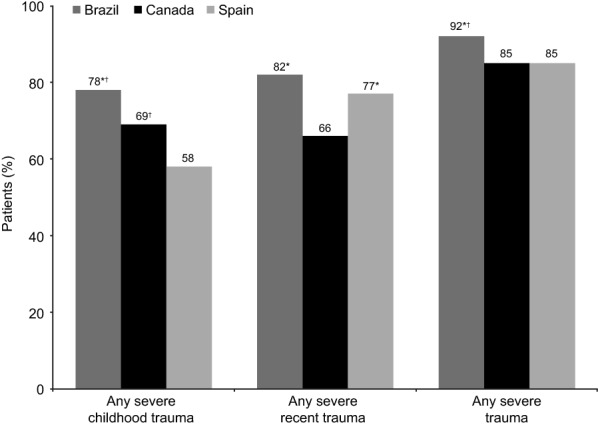
Proportion of patients reporting severe trauma by country (patient-reported cohort). Severe trauma = score of 6 or 7 on a 7-point severity scale. *p ≤ 0.05 vs Canada; ^†^*p* ≤ 0.05 vs Spain

A total of 658 patients reported severe trauma (88% of the overall patient-reported cohort; 68% severe childhood trauma and 75% severe recent trauma). In all, 419 patients (56%) reported both severe childhood and recent trauma (Table 1). As shown in Fig. 1, the proportion of patients reporting severe trauma was significantly higher among those in the acute versus the remission phase of depression across all trauma categories (all *p* < 0.01). The proportion of patients reporting severe trauma was higher in Brazil than in Canada and Spain across all categories (Fig. 2; most differences between Brazil and other countries, *p* ≤ 0.05).

**Fig. 3:**
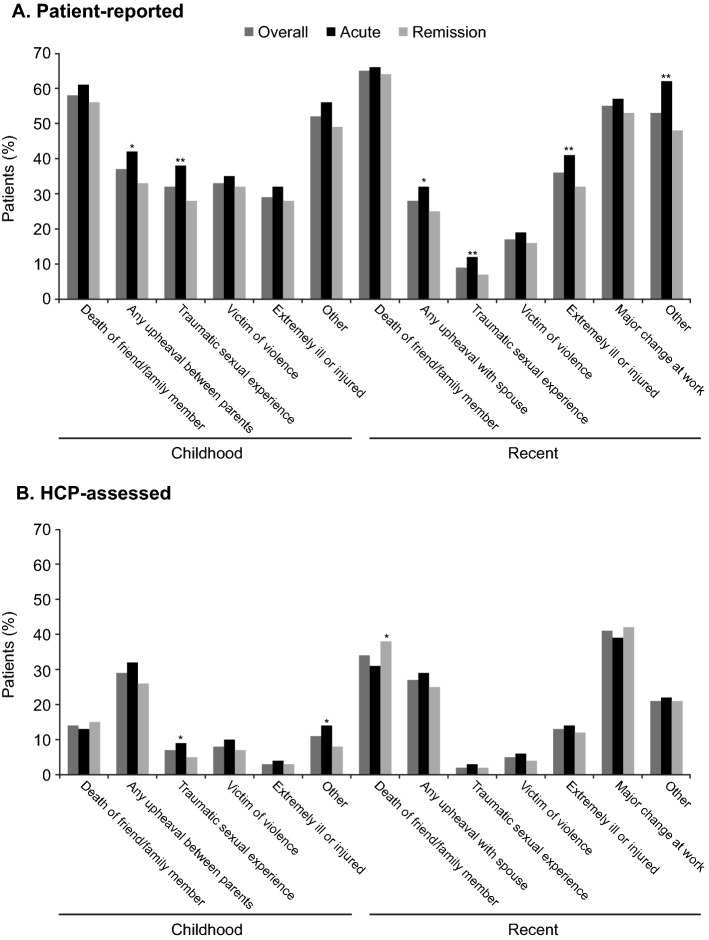
Proportion of patients reporting childhood and recent traumatic experiences: **A** patient-reported cohort and **B** HCP-assessed cohort (overall and by phase of depression). *p < 0.05, **p < 0.01 vs remission phase. HCP, healthcare provider

The most common childhood traumatic experience reported by patients was the death of a very close friend or family member (reported by 61% of patients in the acute phase of depression and 56% of those in remission) (Fig. 3). Patients in the acute phase of depression were significantly more likely than those in the remission phase to report any upheaval between their parents (42% vs 33%; *p* < 0.05) and childhood sexual abuse (38% vs 28%; *p* < 0.01). Overall, 33% of patients reported having been a childhood victim of violence (35% in the acute phase of depression and 32% in remission). HCPs reported lower rates of all types of traumatic events during childhood than patients (Fig. 3).

The most common recent traumatic experiences reported by patients were the death of a very close friend or family member (65%) and a major change at work (55%) (Fig. 3). According to HCPs, however, only 34% of patients had recently experienced bereavement and 41% had experienced a major change at work. Patients in the acute phase of depression were significantly more likely to report the following recent traumatic events compared with patients in remission: extreme illness or injury (41% vs 32%, respectively; *p* < 0.01), any upheaval with spouse (32% vs 25%; *p* < 0.05), traumatic sexual experience (12% vs 7%; *p* < 0.01), and other recent traumatic experiences (62% vs 48%; *p* < 0.01).

**Fig. 4:**
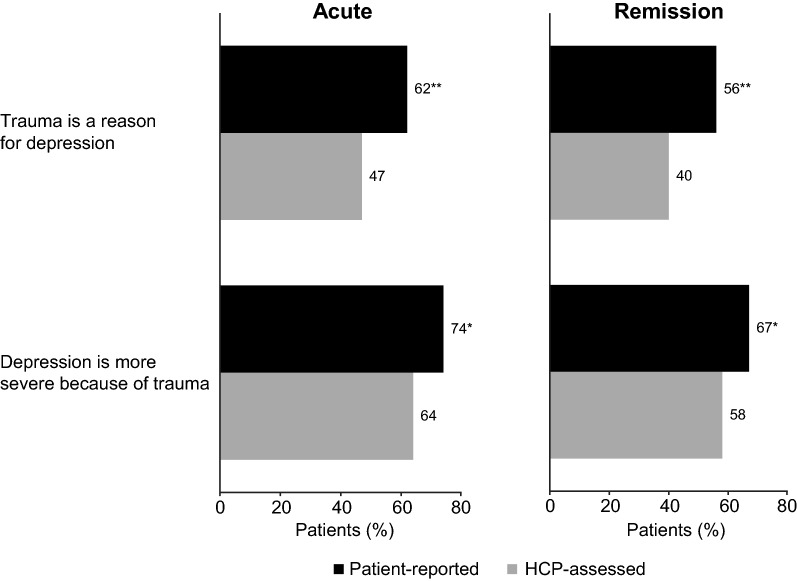
Proportion of patients and HCPs who considered that the patient’s experience of trauma was a reason for their depression and that their depression was more severe because of trauma (by phase of depression).*p < 0.05, **p < 0.01 vs HCP-assessed cohort. HCP, healthcare provider

Irrespective of the phase of depression, patients were more likely than HCPs to feel that their experience of trauma had contributed to their/the patient’s depression (58% vs 43%, respectively; *p* < 0.01) and that the depression was more severe because of their/the patient’s experience of trauma (70% vs 61%; *p* < 0.01) (Fig. 4).

### ODQ and FAST scores

Mean total ODQ scores were significantly higher (i.e., indicative of more severe emotional blunting) in patients with a history of severe trauma than in those without severe trauma in each trauma category (all *p* < 0.01) (Table 2). For ODQ domain scores, the greatest differences between patients with and without severe trauma were seen for the ‘reduction in positive emotions’ (difference for each severe trauma category, *p* < 0.01), ‘general reduction in emotions’ (difference, *p* < 0.05 for severe childhood trauma and *p* < 0.01 for severe recent and any severe trauma), and ‘not caring’ domains (difference, *p* < 0.05 for any severe trauma and *p* < 0.01 for severe childhood or severe recent trauma). No significant differences were seen between groups for the ‘antidepressant as cause’ domain in any trauma category. Mean total FAST scores were significantly higher (i.e., worse) in patients with a history of severe childhood or recent trauma than in those without (both *p* < 0.01) (Table 2).

**Table 2:** Mean (SD) ODQ total scores, ODQ domain scores, and FAST total scores in patients with/without severe trauma by trauma type (patient-reported cohort)

	Severe childhood trauma		Severe recent trauma		Any severe trauma	
	Yes (*n* = 515)	No (*n* = 237)	Yes (*n* = 562)	No (*n* = 190)	Yes (*n* = 658)	No (*n* = 94)
ODQ total score	90.8 (18.1)**	86.1 (18.3)	90.6 (18.0)**	85.6 (18.7)	90.1 (18.3)**	83.9 (17.3)
Reduction in positive emotions	20.7 (4.3)**	19.7 (4.4)	20.8 (4.2)**	19.4 (4.6)	20.6 (4.3)**	19.2 (4.5)
General reduction in emotions	18.3 (3.8)*	17.6 (3.8)	18.4 (3.8)**	17.3 (4.0)	18.3 (3.8)**	16.7 (3.8)
Not caring	17.9 (4.6)**	16.8 (4.7)	17.9 (4.7)**	16.6 (4.5)	17.7 (4.7)*	16.5 (4.4)
Emotional detachment from others	15.8 (5.8)*	14.7 (5.7)	15.6 (5.8)	15.0 (5.7)	15.5 (5.8)	14.8 (5.5)
Antidepressant as cause	18.0 (6.2)	17.3 (5.9)	18.0 (6.0)	17.2 (6.3)	17.9 (6.1)	16.7 (6.2)
FAST total score	40.2 (17.1)**	35.7 (17.4)	40.6 (17.3)**	33.4 (16.1)	39.2 (17.4)	35.6 (16.4)

*FAST* Functioning Assessment Short Test, *ODQ* Oxford Depression Questionnaire, *SD* standard deviation

^*^p < 0.05  vs ‘No’ cohort

^**^p < 0.01 vs ‘No’ cohort

### Relationship between trauma and emotional blunting

**Fig. 5:**
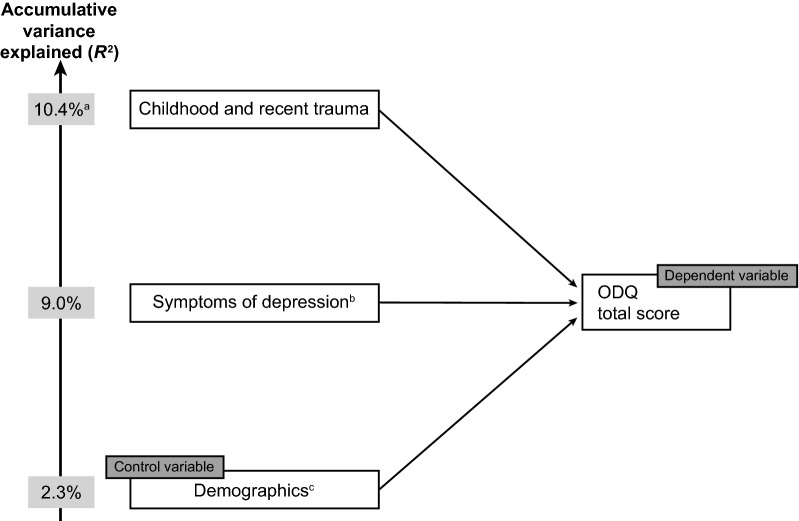
Stepwise multivariate regression analysis to predict the extent to which the ODQ score is predicted by severe trauma.^a^ODQ percentage is accumulative, reflecting history of trauma and control variables, ^b^anxiety, mood symptoms (sadness, lack of enjoyment, hopelessness), physical symptoms (decrease in weight or appetite, disturbed sleep, fatigue, sexual dysfunction), and cognitive symptoms (trouble concentrating, difficulties making plans, forgetfulness) were assessed by the survey questionnaire, ^c^age group, sex, education, and country. ODQ, Oxford Depression Questionnaire

In the multivariate regression analysis, symptoms of depression (specifically mood symptoms, followed by physical symptoms) were found to account for the greatest part of the accumulative variance in ODQ total score (Fig. 5). Experiencing both severe childhood and recent trauma was found to statistically significantly impact the ODQ total score (*p* = 0.001), although it accounted for only 1.4% of the total accumulative variance in ODQ total score (*R*^2^, 10.4%). Experiencing either severe childhood or recent trauma alone had no significant impact on ODQ total score.

## Discussion

Our findings show that experience of psychological trauma is prevalent in patients with depression; almost all patients (97%) reported experiencing a traumatic event at some point during their life, and 88% reported severe trauma. The prevalence of self-reported trauma in this study is higher than that reported in other populations with depression [7, 21, 43]. An analysis of general population surveys from 24 countries across six continents, with 68,894 adult respondents, found that more than 70% of respondents had experienced at least one traumatic event [44]. Five types of trauma (witnessing death or serious injury, the unexpected death of a loved one, being mugged, being in a life-threatening automobile accident, and experiencing a life-threatening illness or injury) accounted for more than half of all exposures. In the current study, the proportion of patients in Brazil reporting severe trauma was higher across all categories than in Canada and Spain; geographic differences in the prevalence of trauma are not unexpected [44], and may be due to factors such as differences in willingness to disclose sensitive information and differing levels of violence in society.

In the current study, more than two-thirds of patients reported experiencing severe childhood trauma and three-quarters reported severe recent trauma. Patients who had experienced severe trauma reported more severe emotional blunting (i.e., higher ODQ total score) and greater functional impairment (i.e., higher FAST total score) than those who had not experienced severe trauma. In the multivariate regression analysis, experiencing both severe childhood and recent trauma was found to have a significant and independent impact on severity of emotional blunting (i.e., higher ODQ total score).

It is therefore noteworthy that our findings suggest that HCPs may underestimate the proportion of patients with depression who have experienced psychological trauma, especially during childhood. Furthermore, patients were significantly more likely than HCPs to think that psychological trauma was a reason for their depression and that their depression was more severe because of it. Other studies have shown clinically relevant differences between patient and HCP perceptions of depression [45–47]. Nevertheless, the possibility that patients may have overestimated the potential impact of their experience of psychological trauma on their depression cannot be excluded.

An improved understanding of patients’ lived experiences of depression is important in order to ensure that treatment is appropriate to their needs. Patients with a history of trauma appear to have a suboptimal response to treatment with some antidepressants [7, 23]. Improved recognition of patients with trauma and emotional blunting may permit more targeted therapeutic interventions, resulting in improved treatment outcomes. For example, the multimodal antidepressant vortioxetine has demonstrated significant efficacy in relieving depressive and anxiety symptoms in patients with MDD reporting childhood or recent trauma [43]. Furthermore, if patients experience blunted emotions with their current treatment for depression then alternative antidepressants should be explored. In one recent study, patients with MDD who experienced emotional blunting and an inadequate response to selective serotonin reuptake inhibitor or serotonin-noradrenaline reuptake inhibitor monotherapy reported significant improvements in emotional blunting, depressive symptoms, cognitive performance, motivation and energy, and overall functioning after 8 weeks of treatment with vortioxetine 10–20 mg/day [48].

### Methodologic considerations

The main strengths of this study are its large sample size, with participants recruited from three countries, and assessment from the perspective of both patients and HCPs. Potential limitations include selection bias (i.e., by design all patients were experiencing emotional blunting and patients may have been more likely to consent to study participation because of their experience of trauma) and the possibility of recall bias with retrospective reporting of childhood trauma, particularly in individuals with mental health disorders [49, 50]; for example, depressed mood may increase the likelihood of recall of negative experiences. It should also be noted that data are lacking concerning duration and frequency of exposure to the traumatic experience(s) and history of any other psychiatric disorders, particularly post-traumatic stress disorder, as these factors may also potentially have contributed to the prevalence and severity of emotional blunting in the two patient cohorts. Finally, comparisons between the self-assessed and HCP-assessed cohorts should be interpreted with caution, as patient- and HCP-provided responses were unmatched (i.e., the patients who completed the survey themselves were not the same patients as those reported on by HCPs).

## Conclusion

In summary, our study shows that a very high percentage of patients with depression and emotional blunting self-report exposure to childhood and/or recent psychological trauma, and that emotional blunting is more severe in patients with depression who have experienced severe psychological trauma than in those who have not. While patients often reported that trauma is a reason for their depression and that their depression is more severe because of this experience, exposure to potential psychological trauma and its possible impact in patients with depression appeared to be under-recognized by HCPs. Differences between patient and HCP perspectives concerning the experience of emotional blunting in MDD will be further explored in the final paper in this series [51]. Improved recognition of patients who have experienced psychological trauma and are experiencing emotional blunting may permit more targeted therapeutic interventions, potentially resulting in improved treatment outcomes.

## Data Availability

The datasets presented in this article are not readily available given the informed consent provided by survey participants. Requests to access the datasets should be directed to the authors.
